# Deciphering the rhizosphere bacteriome associated with biological control of tobacco black shank disease

**DOI:** 10.3389/fpls.2023.1152639

**Published:** 2023-04-03

**Authors:** Yi-Nan Ma, Yi-Lin Gu, Jing Liu, Yuqin Zhang, Xinwei Wang, Zhenyuan Xia, Hai-Lei Wei

**Affiliations:** ^1^ Key Laboratory of Microbial Resources Collection and Preservation, Ministry of Agriculture and Rural Affairs, Institute of Agricultural Resources and Regional Planning, Chinese Academy of Agricultural Sciences, Beijing, China; ^2^ Zunyi Tobacco Company of Guizhou Provincial Tobacco Corporation, Zunyi, China; ^3^ China National Tobacco Corporation Shandong Branch, Jinan, China; ^4^ Key Laboratory of Tobacco Pest Monitoring & Integrated Management in Tobacco, Tobacco Research Institute of Chinese Academy of Agricultural Sciences, Qingdao, China; ^5^ Yunnan Academy of Tobacco Agricultural Science, Kunming, China

**Keywords:** tobacco black shank, amplicon sequencing, biocontrol agent, microbiome, co-occurrence network

## Abstract

**Introduction:**

The black shank disease seriously affects the health of tobacco plants. Conventional control methods have limitations in terms of effectiveness or economic aspects and cause public health concerns. Thus, biological control methods have come into the field, and microorganisms play a key role in suppressing tobacco black shank disease.

**Methods:**

In this study, we examined the impact of soil microbial community on black shank disease basing on the structural difference of bacterial communities in rhizosphere soils. We used Illumina sequencing to compare the bacterial community diversity and structure in different rhizosphere soil samples in terms of healthy tobacco, tobacco showing typical black shank symptoms, and tobacco treated with the biocontrol agent, Bacillus velezensis S719.

**Results:**

We found that Alphaproteobacteria in the biocontrol group, accounted for 27.2% of the ASVs, was the most abundant bacterial class among three groups. Heatmap and LEfSe analyses were done to determine the distinct bacterial genera in the three sample groups. For the healthy group, Pseudomonas was the most significant genus; for the diseased group, Stenotrophomonas exhibited the strongest enrichment trend, and Sphingomonas showed the highest linear discriminant analysis score, and was even more abundant than Bacillus; for the biocontrol group, Bacillus, and Gemmatimonas were the largely distributed genus. In addition, co-occurrence network analysis confirmed the abundance of taxa, and detected a recovery trend in the network topological parameters of the biocontrol group. Further functional prediction also provided a possible explanation for the bacterial community changes with related KEGG annotation terms.

**Discussion:**

These findings will improve our knowledge of plant-microbe interactions and the application of biocontrol agents to improve plant fitness, and may contribute to the selection of biocontrol strains.

## Introduction

Tobacco black shank (TBS) disease, caused by *Phytophthora parasitica* var. *nicotianae* (Breda de Haan), is one of the most damaging and extensive diseases of tobacco (Kannwischer and Mitchell, 1978). It was first reported in Indonesia by Van Breda de Haan in 1896 and spread to China in 1950 (Breda de Haan, 1896). *P. parasitica* var. *nicotianae was* mainly consisted of four races in terms of race 0, 1, 2 and 3. Race 0 and 1 has been reported in China and the black shank disease in Yunnan is mainly caused by race 0 (Apple, 1962; Liu et al., 2016). Management strategies for the prevention and suppression of TBS include crop rotation, resistant cultivars, and chemical control. However, only very few of these control measures are sufficiently effective or economical (Kong et al., 1995; Gallup et al., 2006). Additionally, *P. parasitica* var. *nicotianae* have shown different levels of resistance to some commercial chemical fungicides in many places in China. Nevertheless, the overuse of chemicals is a major concern for agriculture and public health (Munita and Arias, 2016). For guaranteed production of organic, green, and pollution-free tobacco leaves, the development and application of other non-chemical technologies are necessary for successful prevention and control of TBS.

In the agricultural industry, biological control is a choice to deal with crop diseases. Compared to chemical agents, especially traditional fungicides, biocontrol agents have several advantages, such as low selective pressure, no pesticide residue, and improved soil microbial structure. Attempts to apply biological control methods to prevent TBS have been reported, and the results have turned out to be promising. Nonpathogenic binucleate fungus *Rhizoctonia* (BNR) were used to control TBS at the seedling stage under greenhouse conditions, and 70% effectivity was achieved (Cartwright and Spurr, 1998). *Bacillus subtilis* Tpb55 showed a promising controlling effect of 70.66% on TBS under pot experimental conditions (Han et al., 2016). *Trichoderma* strains AR-4, Tv-1, and ST4-1 isolated from plant’s stems, roots, and rhizosphere soil showed a more than 60% effectiveness on TBS (Liu et al., 2022). The mechanisms of action of the biological agents on TBS were also investigated. *Bacillus velezensis* Ba168 damages the cell walls and membranes of *P. parasitica* (Guo et al., 2020). However, there are few reports on the analysis of how black shank pathogen affect the microbiome structure in rhizosphere of tobacco. Thus, we utilized a previously isolated bacterial strain *Bacillus velezensis* S719 from tobacco rhizosphere with antagonism to TBS to explore the consequences of applied biocontrol agent on the rhizospheric microbiome.

The concept of “holobiont” indicates the entirety of the host plant and its microbiota (Zilber-Rosenberg and Rosenberg, 2008; Vandenkoornhuyse et al., 2015). Plants provide nutrition and niches for the growth and colonization of microbes, while microbial activities influence plant health through multiple mechanisms (Philippot et al., 2013; Huang et al., 2014). Plants can adapt to most biotic and abiotic adversities with the help of microbes, which aid their survival and defense against pathogens. In this phenomenon, rhizosphere microbes play the most critical roles (Berendsen et al., 2012; De Zelicourt et al., 2013). Researches have shown that microorganisms could improve plant health in many aspects through inhibited growth of pathogens, activated plant immunity, induced resistance, promoted nutrient absorption and plant growth, adaptation to environmental changes, and promoted establishment of microbial communities (Philippot et al., 2013; Qu et al., 2020). Simultaneously, a “crying for help” mechanism is adopted by plants facing the threat of pathogen invasion, which is associated with the selective enrichment of beneficial microorganisms (Yi et al., 2011; Rolfe et al., 2019).

The recent application and easy access to high-throughput omics technologies allowed a detailed dissection of the complex plant-associated microbial communities, which offered an extended understanding of microbiome structures and their interactions with plants (Berg et al., 2016; Jansson and Baker, 2016; Bakker et al., 2020). Here, we conducted a comprehensive structural dynamic profiling of the rhizosphere bacterial communities of healthy, black shank infected, and biocontrol agent-treated tobacco plants to answer the following questions: (1) How do different conditions affect the composition of rhizosphere bacterial communities? (2) Do any key taxa play crucial roles in community changes during external treatment?

## Materials and methods

### Soil sample management

The soils used in this study were collected from Yunnan Province, China in 2019. Sampling locations were in Yuxi City (24.145°N, 102.476°E). There were two sets of fields, both of which were loam, one was used for black shank disease studies all year round and the other is a healthy control field. The tobacco rhizosphere soil samples obtained from the diseased field was regarded as the disease group (D group), the samples obtained from the healthy control field were regarded as the healthy group (H group), which were 600 m away from the diseased field, and the biocontrol group (B group) was defined as the diseased field treated with biocontrol agent consisting of *Bacillus velezensis* S719 after tobacco seedlings were transplanted. The *Bacillus velezensis* S719 was fermented with the media at 29-31°C for 48 h at a speed of 150 rpm with 10% ventilation, 0.2% sodium humate, then added 0.1% chitosan and 0.1% chitin. The forming agent was applied during transplanting with a dosage of 210-330 g/km^2^. The variety of tobacco was Yunyan 99.

Samples were collected during the peak period of black shank disease, which was about 8 weeks after the transplanting in July. The zig-zag random sampling method was adopted and eight tobacco plants mixed as one rhizosphere soil replicate (Smith and Rowe, 1984). There were 5 replicates of each treatment. The whole root system of tobacco was dig out and shaken vigorously to remove the loosely bound soil, and the tightly adhered soil (about 1-2 mm) was kept in a self-sealing bag as the final rhizosphere soil sample. The samples were put in a freezer, brought back to the laboratory, and stored at -80°C for further processing. The physical and chemical attributes of the soils were measured but no significant difference was observed (data not shown).

### DNA preparation and *16S rRNA* gene illumina sequencing

Total genomic DNA was extracted from the soil using a FastDNA Spin Kit for Soil (MP, USA), according to the instructions provided by the manufacturer. Briefly, 0.25 g fresh soil samples were ground with lysis buffer using the Lysing Matrix E tube for 30 min, and subsequently purified with inhibitor removal buffer, binding buffer, and wash buffer in the filtered tubes. Afterwards, the DNA was examined using 1.0% (v/v) agarose gel electrophoresis and quantified using a Nanodrop One Spectrophotometer (Thermo Scientific, USA).

For the *16S rRNA* gene libraries, the V3-V4 region was amplified using the universal primers 341F (5’-CCTAYGGGRBGCASCAG-3’) and 806R (5’-GGACTACHVGGGTWTCTAAT-3’) (Roggenbuck et al., 2014). Amplification was performed using the following PCR program: 95°C for 3 min, followed by seven cycles of 95°C for 45 s, 65°C for 1 min (decreasing at 2°C per cycle), and 72°C for 90 s; second, 30 cycles of 95°C for 15 s, 50°C for 30 s, and 72°C for 30 s; and finally, 72°C for 5 min as a final extension. PCR amplification was performed using a Phanta Max Master Mix Kit P515 (Vazyme, China). After 1.0% (v/v) agarose gel electrophoresis, samples were sent to Novogene Co., ltd for high-throughput sequencing on the Illumina P300 platform.

### Raw data assembly and dimension reduction analysis

The resulting sequences provided by the company were demultiplexed and quality-filtered using Vsearch on the Galaxy platform (version 2.7.2) (Afgan et al., 2018). An amplicon sequence variant (ASV) table was generated using DADA2 (version 1.8) (Callahan et al., 2016). After removing ASVs not present in at least 5% of all samples or less than 0.1% of the total abundance, we identified 2,743 ASVs of bacterial and archaea for subsequent analysis excluding ASVs taxonomically classified as mitochondria or chloroplasts. The taxonomy of each sequence was analyzed by RDP Classifier (Wang et al., 2007) against the SILVA Small Subunit rRNA database (Version 138) (www.arb-silva.de/documentation/release-138/) using a confidence threshold of 0.7 (Quast et al., 2012). Downstream analysis was performed using MicrobiomeAnalyst online pipeline (Version 2.0) Marker Data Profiling (MDP) and Shotgun Data Profiling (SDP) modules (https://www.microbiomeanalyst.ca/) (Dhariwal et al., 2017). All samples were rarefied to the sample with the least number of sequences (48,602 read counts) prior to downstream analyses.

### Bioinformatic analysis

#### Diversity analysis

Alpha analysis was carried out based on Chao1 and Shannon diversity indices at the ASV and genus levels. Beta analysis was done using Bray-Curtis dissimilarity at the ASV or genus level.

#### Taxonomic composition

**Figure 1:**
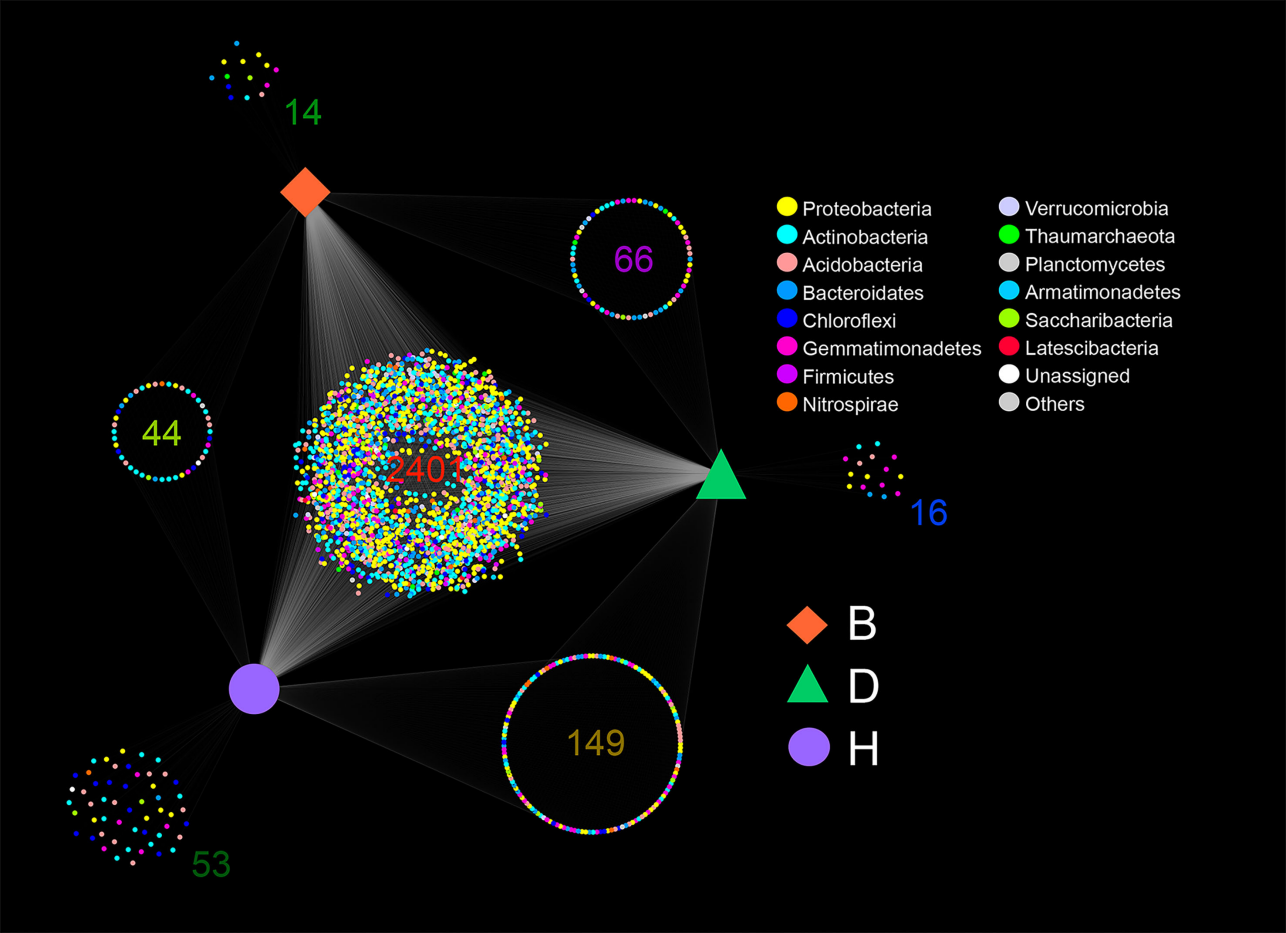
Amplicon sequence variants (ASVs) shared or monopolized by different samples. Color of the nodes representing the bacterial phyla, and orange square, green triangle and purple circle representing the three groups B (Biocontrol agent), D (Disease) and H (Healthy). The number of ASVs is provided for each cluster.

The taxon composition was generated at the phylum, class and genus levels. As the phylum level Proteobacteria always accounted for the highest proportion of bacterial taxa, we analyzed the class and genus levels for the “Relative abundance of bacterial taxa” section (**Figure 1**). A condition of minimum count = 2, prevalence in samples > 10%, and merging mall taxa with counts < 10 were used. Only the top 15 taxa were listed.

#### Analysis of the differential taxa

A heat map was generated using the Euclidean distance measure and the Ward clustering algorithm at the genus level. The linear discriminant analysis (LDA) effect size (LEfSe) method was used to reveal the microbial taxa differences present among different soil groups by a LDA score > 2 and p< 0.05 (Segata et al., 2011).

#### Network construction

Correlation of co-occurrence network analysis was calculated using integrated Network Analysis Pipeline (iNAP) (Feng et al., 2022). SparCC method was used to calculate the network with the following parameters: correlation strengh exclusion threshold = 0.1, 100 shuffled times, threshold value > 0.9 and *p*-value = 0.05. The outcome data was investigated using Gephi software (Version 0.9.2)(Bastian et al., 2009).

#### Function annotation and prediction

Functional prediction was performed using PICRUSt2 module in MDP based on Greengene database (Version 13_8) with the default settings (Douglas et al., 2020).

### Statistical analyses

Statistical analyses were performed using IBM SPSS Statistics 17.0 (SPSS, Chicago, IL, USA) or GraphPad 9.0 (GraphPad Software, La Jolla, CA, USA).

## Results

### Bacterial community compositions differing with different treatments

All samples used in this study are grouped into three categories (**Supplementary Table 1**). The rhizosphere soil from tobacco plants showing clear black shank symptoms, named Ph1-5A, was grouped as Disease (D); the rhizosphere soil associated with healthy plants, named Ph1-5D, was grouped as Healthy (H); and that from the plants treated with biocontrol agents was named ANS1-5, and grouped as Biocontrol (B). The disease index was calculated about 8 weeks after the transplanting and a significant difference was observed with the highest in D group and the lowest in H group (**Supplementary Figure 1**). The total number of amplicon sequence variants (ASVs) generated from 15 samples was 23,517. After filtering the three sampling groups, a total of 2743 high-quality ASVs were kept with a library size ranging from 48,602 to 55,226. The rarefaction curves flattened after the sequence size exceeded 30,000, indicating sufficient sequencing depth (**Supplementary Figure 2**).

In general, most of the ASVs were shared by the three groups with 2,041 ASVs distributed in 13 bacterial phyla and one archaea phyla, comprising 80.83% of the B group ASVs, 77.54% of the D group ASVs, and 77.11% of the H group ASVs, respectively (**Figure 1** and **Supplementary Table 2**). The H group had the most unique ASVs (2%, 53 out of 2,647) among the three groups, whereas the B and D groups had much fewer unique ASVs (0.55%, 14 out of 2,525 and 0.6%, 16 out of 2,632) (**Figure 1** and **Supplementary Table 2**). More ASVs were found in D-H shared groups (5.46%, 149 out of 2,729) than in B-H shared groups (1.61%, 44 out of 2,727) and B-D shared groups (2.45%, 66 out of 2,690) (**Figure 1** and **Supplementary Table 2**). Interestingly, the percentage of Proteobacteria in group D only ranked fourth, while in the other groups, this phylum ranked the top two. In group D, the phylum Actinobacteria accounted for 24.5% (645 out of 2,632) of all ASVs at the top of the list. This phylum in the other two groups accounted for only 7.1% (179 out of 2,525, group B) and 12.5% (331 out of 2,647, group H), respectively (**Figure 1** and **Supplementary Table 2**).

### Comparative structure of bacterial community in different soil samples

**Figure 2:**
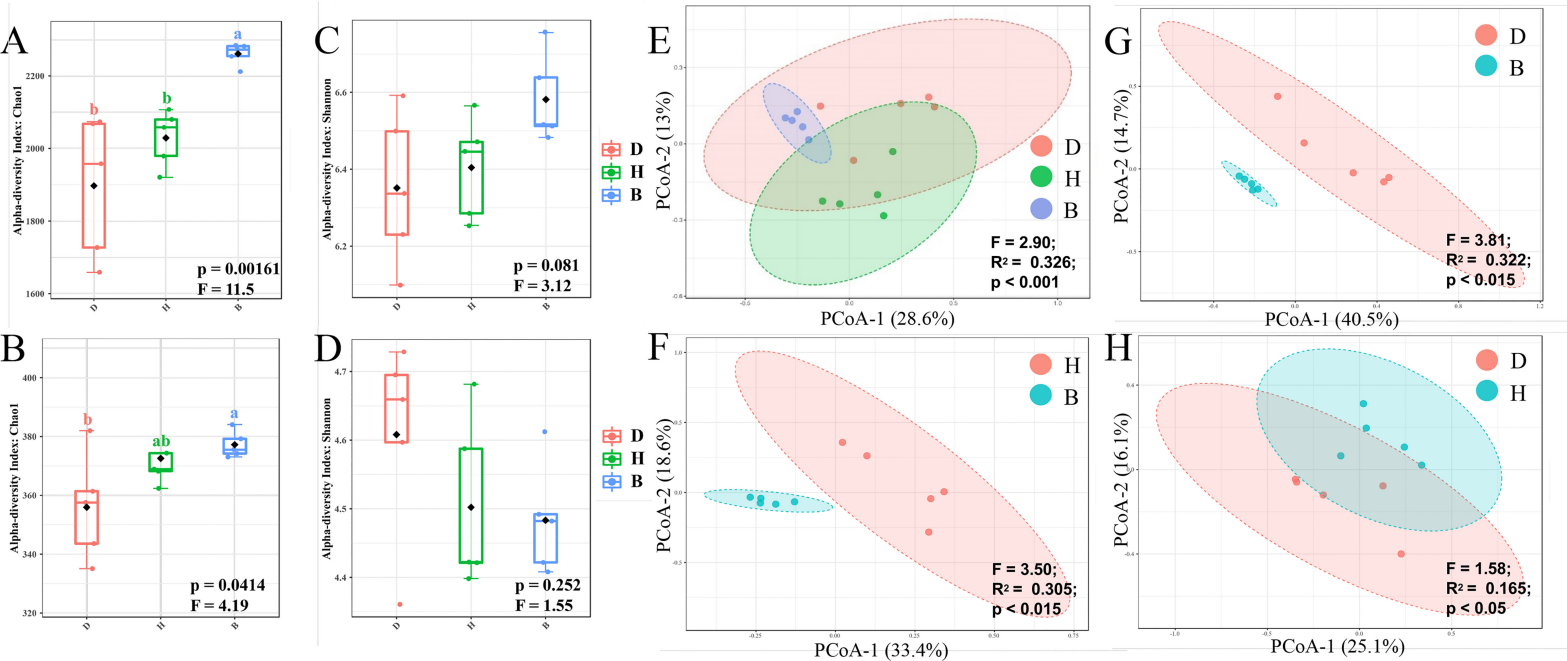
Diversity analyses of bacterial community in soil samples. Alpha diversity indicated by Chao1 **(A)** and Shannon **(B)** index of ASV level, and Chao1 **(C)** and Shannon **(D)** index of genus level was visualized by box plots. Among the estimated values ranging between 25% and 75%, of which the median was shown, the minimum and the maximum observed values within each dataset were listed. The p values across different groups were displayed at the bottom of each graph. Statistically significant differences (One-way ANOVA) were calculated between different soil samples. Lower letters represented statistical differences at the 95% confidence interval (p < 0.05). Beta diversity indicated by Bray-Curtis dissimilarity was visualized by PCoA method on ASV level across three different groups **(E)**, H and B **(F)**, D and B **(G)**, D and H **(H)**. Statistically significant differences (PERMANOVA) were calculated between different groups, F-value, R square and p-value were labeled.

To evaluate the diversity of the microbial communities within a single sample, alpha diversity was calculated by Chao1 and Shannon index. Group B samples were more diverse than groups D and H samples at the ASV level (**Figure 2A**, **Supplementary Table 3**), indicating an increase in ASV richness and evenness with the supplementation of biocontrol agents. The genus level richness pattern revealed by the Chao1 index remained constant with ASV level, with group B being significantly higher than group D (**Figure 2B**, **Supplementary Table 3**). However, Shannon index (**Figures 2C, D**, **Supplementary Table 3**) at both ASV and genus level showed no significant difference within groups D, H and B. The microbial communities in groups D and H were very similar in terms of alpha diversity, with minimal differences revealed by the Chao1 and Shannon indices.

Unconstrained principal coordinates analysis (PCoA) was performed using the Bray-Curtis dissimilarity showing a clear separation between groups B, D, and H across the first axis, which explained 28.6% of the ASV level (**Figure 2E**). Further comparisons between every two groups were subsequently conducted. The clear difference between groups H and B (**Figure 2F**) or D and B (**Figure 2G**) can be observed (*p*-value < 0.015). Treatment with biocontrol agents had a major influence on microbiome composition, as evidenced by the relatively high R^2^ and low *p*-value in groups H and B (**Figure 2F**), D and B (**Figure 2G**) comparing with groups D and H (**Figure 2H**).

**Figure 3:**
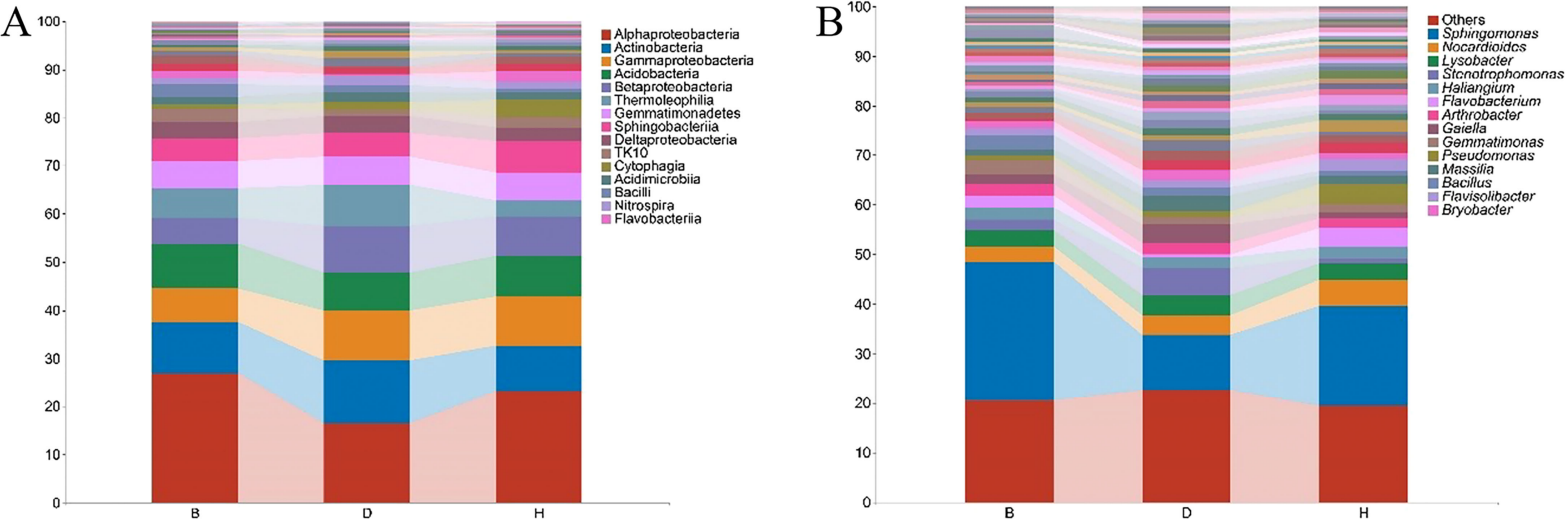
Relative abundance of bacterial taxa at the class **(A)** and genus **(B)** levels. Small taxa were merged into others with the ASV counts less than 10 for class level. Only top 50 taxa were calculated for genus level and the rest was merged into others. Bar charts were used for visualization with top 15 items labeled.

To better understand the microbial composition between the sampling groups, the relative abundance of the bacterial taxa was calculated at the class and genus levels. We detected 51 bacterial classes with merging small taxa into “others” (ASV counts less than 10) (**Figure 3A**, **Supplementary Table 4**). Among these, Alphaproteobacteria was the most prevalent bacterial class, accounting for > 15% of the ASVs in each sampling groups. In B groups, Alphaproteobacteria accounted for 27.2% of the ASVs, at least two folds more than other terms in the same group; Actinobacteria was abundant in group D, accounting for 13% of the ASVs, while the proportions in group B and H were 10.6% and 9.5%, respectively; Sphingobacteria and Cytophagia were abundant in H group, accounting for 6.7% and 3.5% of the ASVs, respectively. Further structural details of microbiome are revealed at the genus level (**Figure 3B**, **Supplementary Table 4**). For instance, *Sphingomonas* was the most abundant genus, accounting for 27.9%, 11% and 20% of the ASVs in B, D and H groups, respectively. *Stenotrophomonas* reached to 5.5%, but *Flavobacterium* decreased to 0.5% of the ASVs in group D. The proportion of *Bacillus* enriched to 2.9% in group B and *Pseudomonas* was abundant in group H samples (4% of the ASVs) (**Supplementary Table 4**).

**Figure 4:**
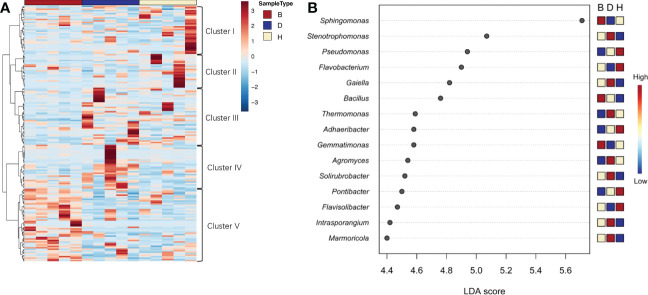
Differentially abundant bacterial genera in different soil groups. **(A)** Heatmap analysis showed group specific prevalence of clusters. Distance measure was Euclidean and Ward clustering algorithm was used. **(B)** Graphical summary of LEfSe analysis. Significant taxa were ranked in decreasing order by their linear discriminant analysis (LDA) scores (x axis). The mini heat map on the right of the plot indicated whether the taxa were higher (red) or lower (blue) in each group.

To further investigate the effects of soil state and biocontrol agents on microbial diversity, differentially abundant genera were investigated. In group B communities, the enriched ASVs were primarily attributed to cluster V, whereas other clusters only had one or two samples among the five from each treatment showed distinct difference with other samples (**Figure 4A**). Moreover, a clear prevalence of cluster II was abundant in group H communities. LEfSe analysis was applied to further uncover the taxon distribution among different groups (**Figure 4B**). The most differentially abundant genus was *Sphingomonas*, which relative abundance was elevated by supplementation with biocontrol agents and reduced when the plants suffered from pathogen infection. According to the heat map results on the right, the most frequent genera in group H were *Pseudomonas*, *Flavobacterium*, *Adhaeribacter, Pontibacter*, and *Flavisolibacter*. Group D samples were specifically enriched *in Stenotrophomonas, Galella, Thermomonas, Agromyces, Solirubrobacter, Intrasporangium, and Marmoricola.* Contrastingly, *Sphingomonas, Bacillus*, and *Gemmatimonas* were largely distributed in group B samples, indicating a distinct microorganism difference between sampling groups.

### Different co-occurrence network patterns in different groups

**Figure 5:**
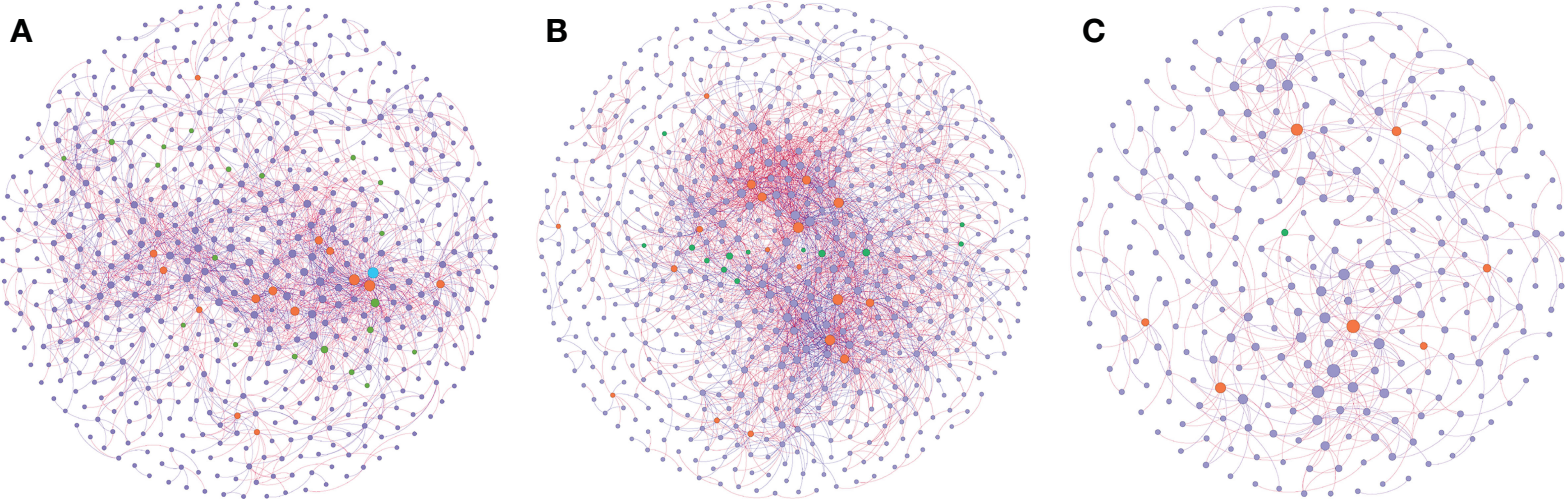
Co-occurrence network inference of the rhizosphere microbiomes compared. Co-occurrence index based on SprCC method correlation of *16S rRNA genes* (ASV level) were extracted from soil samples in three conditions: healthy soil **(A)**, disease soil **(B)** and the soil supplemented with biocontrol agent **(C)**. Correlation was calculated using iNAP online pipeline “SprCC”, with correlation strength exclusion threshold = 0.1, 100 shuffled times, threshold value > 0.9 and *p* value = 0.05. Each node represented a single ASV and hub nodes were labeled differed with other nodes (module hubs labeled in orange, connectors labeled in green, network hubs labeled in blue and other nodes labeled in purple). A red edge indicated a positive correlation between two individual nodes, while a blue edge indicates a negative correlation.

Network analysis was performed to explore the co-occurrence among microbes and to identify the pattern differences between the sampling groups (**Figure 5**). Through the analysis, a stable network could be constructed from group H, with 619 nodes and 2158 edges, and a relatively balanced positive-negative ratio (58%:42%) (**Figure 5A**, **Supplementary Table 5**). With the plants facing pathogen infection, its network nodes and edges increased to 665 and 3125, and the positive-negative ratio tended to be more positive (68%:32%) (**Figure 5B**, **Supplementary Table 5**). After exposure to biocontrol agents, the network nodes and edges dropped dramatically to 300 and 541, respectively; however, the positive-negative ratio remained similar to that in the disease group (67%:33%) (**Figure 5C**, **Supplementary Table 5**).

Microbial communities usually harbor keystone taxa that can be computationally identified as hubs (module hubs and connectors) based on the within-module degree (*Zi*) and among-module connectivity (*Pi*) of the ASVs in the networks (**Supplementary Figure 2**). The network generated by group H samples showed 35 hub nodes with 20 connectors, 14 module hubs, and 1 network hub; while group D and group B samples generated networks that had connectors and module hubs only (13, 18, and 1, 7, respectively) (**Supplementary Tables 6**-**8**). Group H consisted of nine bacterial phyla, where Proteobacteria was the dominant phylum with a percentage of more than 50%. Composition changed dramatically when plants were infected with pathogens; the proportion of Proteobacteria dropped to 15%, while Actinobacteria became dominant, accounting for 46%, and six phyla were left. In the network treated with biocontrol agents, the recovery of the Proteobacteria proportion was above 50%, while the proportion of Actinobacteria decreased to 12%, with only three bacterial phyla remaining. Furthermore, we subdivided Proteobacteria from the phylum level to the class level in terms of Alphaproteobacteria, Betaproteobacteria, and Gammaproteobacteria (**Supplementary Table 9**). Unsurprisingly, the proportion of Alphaproteobacteria changed dramatically enriching in groups H and B and it decreased in group D, which was consistent with the results of the taxa analysis. These results indicated the significant roles of Actinobacteria and Proteobacteria, especially Alphaproteobacteria, in soil health status.

### Functional profiles of bacterial communities

**Figure 6:**
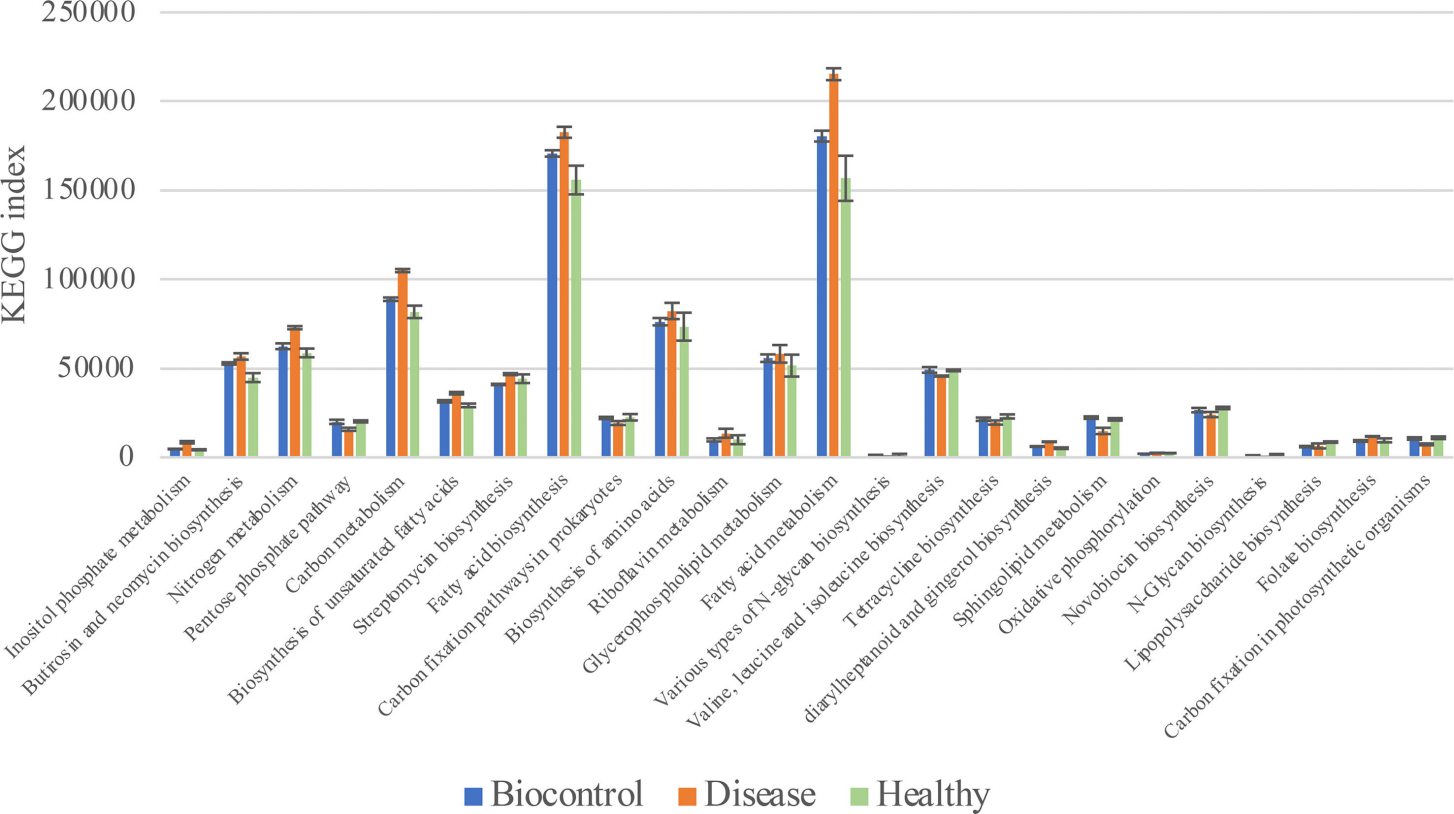
KEGG based bar plot generated by PICRUSt2 indicating significant differences between group D and groups B, H. Green representing the Biocontrol group, red representing the Disease group and blue representing the Healthy group. All the terms listed in the figure have significant difference (Tukey’s test) between group D and B or H.

Phylogenetic Investigation of Communities by Reconstruction of Unobserved States (PICRUSt) was used to predict the metabolic functional spectrum of bacteria (Douglas et al., 2020). After PICRUSt2 prediction and KEGG annotation, we observed a regular trend in the annotated terms. The terms in groups B and H always showed no significant differences, whereas group D showed distinct alterations in their prevalence compared with that in other two groups (**Figure 6**). For detail, “Nitrogen metabolism,” “Carbon metabolism,” “Fatty acid metabolism” and many other secondary metabolisms were upregulated during the black shank disease. The terms “pentose phosphate pathway”, “N-glycan biosynthesis”, “tetracycline biosynthesis, and carbon fixation-related pathways were downregulated in group D compared with that in groups B and H (**Table 1**). In total, there were 14 enriched terms and nine reduced terms in group D.

**Table 1:** KEGG terms significantly changed in the Disease group.

Group D compared to Group B and H	
significantly Up regulated	significantly Down regulated
Inositol phosphate metabolism	Pentose phosphate pathway
Butirosin and neomycin biosynthesis	Carbon fixation pathways in prokaryotes
Nitrogen metabolism	Various types of N-glycan biosynthesis
Carbon metabolism	Valine, leucine and isoleucine biosynthesis
Biosynthesis of unsaturated fatty acids	Tetracycline biosynthesis
Streptomycin biosynthesis	Sphingolipid metabolism
Fatty acid biosynthesis	Novobiocin biosynthesis
Biosynthesis of amino acids	N-Glycan biosynthesis
Riboflavin metabolism	Carbon fixation in photosynthetic organisms
Glycerophospholipid metabolism	
Fatty acid metabolism	
diarylheptanoid and gingerol biosynthesis	
Oxidative phosphorylation	
Folate biosynthesis	

## Discussion

In this study, we investigated the changes in the bacterial community of the *N. tabacum* rhizosphere from the healthy to the diseased stages showing black shank symptoms as well as a stage treated with biocontrol agent. Distinct community changes were observed in many aspects in terms of the diversity index, taxonomy composition, co-occurrence network index, and functional prediction. Several outstanding ASVs in terms of *Sphingomonas Pseudomonas* and *Flavobacterium* genera shaping the rhizosphere microbial structure have been revealed, and significant functional profiles have also been identified in plant-pathogen interactions.

Increasing evidence has revealed that rhizosphere microbes respond to plant root exudates, helping plants absorb nutrients and respond to pathogen invasion (Sun et al., 2021). Lareen et al. reported that plants can recruit specific bacteria and fungi for defense against bacterial wilt in the rhizosphere (Lareen et al., 2016). According to Bakker et al., infection by plant pathogenic fungi resulted in recruitment of specific bacterial groups possessing specific functions that eventually resulted in disease decline. (Bakker et al., 2013). Specific resident plant rhizosphere bacterial communities adapted to plants played important roles in both optimizing growth and protection against pathogens. The recruitment of beneficial microorganisms can also change the physiological functions of plants, allowing them to resist aerial pathogens (Kumar et al., 2012).

The analysis of alpha diversity detected the highest microorganism diversity in group B at both the ASV and genus levels, whereas groups D and H were significantly low. The ASV level result indicated a dynamic balance during disease occurrence, which was consistent with previous reports (Zheng et al., 2021; Yi et al., 2022). The microorganism Chao1 index at the ASV levels increased dramatically due to the addition of biocontrol agents, which was parallel to the report that organic amendments enhance soil microbial diversity and microbial functionality (Shu et al., 2022). However, there was no significant difference in the Shannon index across all soil treatments, suggesting a mild change on the diversity and evenness of microorganism.

Co-occurrence networks have often been applied for the analysis of microorganism correlations (Deng et al., 2012; Su et al., 2020). We observed the most complicated bacterial network consisting of the highest number of nodes and edges in group D compared with groups B and H. However, the stability of the soil structure is not simply defined by the complexity of the networks, which needs to be interpreted from more parameters, such as modularization and connectivity (Deng et al., 2012; Feng et al., 2022). We noticed the dynamic and stable structure of a healthy soil network, which was more diversified than the other two networks (**Figure 4**). Once the plant suffered pathogen invasion, the hub and connection microbes decreased dramatically, and the network structure became simpler than the healthy ones, indicating a manifestation of structural imbalance. With supplementary biocontrol agents, the tight topology of the network was relieved, and the soil structure was directed to a healthy state. A point worth highlighting is the de-modularization ability of the biocontrol agents (**Supplementary Figure 2**). Less hubs or connectors could be observed in group B compared with groups D or H, suggesting the participation of *Bacillus* in the rhizosphere and reshaping the bacterial construction, but we did not determine the population of the introduced strain in the samples, further analysis need to be conducted to supplement and solid the conclusions. Additionally, dynamic amplicon analysis with different sampling periods is necessary for a deeper understanding of this network change.

The differentially abundant taxa in the three experimental groups could potentially play key roles in maintaining the plant health. In the results of heatmap clustering and LEfSe analysis, enriched *Pseudomonas* and *Flavobacterium* genera were observed in the group H rhizosphere soils. *The Pseudomonas* genus consists of a wide range of plant growth-promoting rhizobacteria (PGPR) that have been extensively studied. Inhibitory compounds and siderophores produced by *Pseudomonas* are effective in preventing pathogen invasion (Kloepper et al., 1980; Raaijmakers et al., 2006; Sivasakthi et al., 2014). The genus *Flavobacterium* was also common in disease suppressive soils, reported to play a role in biological control by producing antibacterial factors or substances, extracellular macromolecular degrading enzymes (Kolton et al., 2016; Kwak et al., 2018; Carrión et al., 2019).

The enrichment of *Bacillus* in group B was a consequence of supplementation with the biocontrol agents. Interestingly, the genus *Sphingomonas* was also enriched in group B, with an even higher LDA index than that of *Bacillus* (**Figure 4B**). *Sphingomonas* has been reported to possess multifaceted functions ranging from remediation of environmental contaminants to production of highly beneficial phytohormones, and its biocontrol potential has also been widely reported recently (Innerebner et al., 2011; Qin et al., 2019; Asaf et al., 2020). Additionally, *Bacillus* genera has many promising PGPR as well as antagonistic strain of pathogenic bacteria, with direct antagonistic effect on plant pathogens or work synergistically with other plant beneficial bacterial (Han et al., 2016; Guo et al., 2020; Sun et al., 2022). However, there were few reports on the correlation between *Sphingomonas* and *Bacillus* in plant-microbe interactions. More data are required to know reasons and mechanisms for the effective application of biocontrol agents.

Notably, the phylum Actinobacteria increased dramatically in group D compared with the other groups, which is in accordance with the report by Santos et al., who found that Actinobacteria could be recruited by rice to face extreme conditions and pathogen invasions (Santos-Medellín et al., 2022). Additionally, many microbes have no direct impact on plant fitness, but can modulate community structure and activity by interacting with other microorganisms (Agler et al., 2016; Durán et al., 2018; Sokol et al., 2022).

The functional prediction analysis conducted by PICRUSt2 detected several KEGG terms enriched or reduced in group D, while there was no significant difference between groups B and H. Among which, “Fatty acid biosynthesis” and “Fatty acid metabolism” were the most up-regulated terms in the soil samples from black shank suffered plants, indicating a positive correlation of fatty acid metabolism with disease occurrence. The phylum Actinobacteria has been reported to have diverse fatty acid biosynthesis enrichment potential, especially *Mycobacterium* possessing two fatty acid synthase (FAS) systems (Gago et al., 2011). Furthermore, FAS has also been reported to be related to antimicrobial activities in Actinobacteria, which supports the phenomenon that Actinobacteria are prevalent in plants face biotic or abiotic pressures (Gago et al., 2011). Riboflavin metabolism is responsible for vitamin B2 synthesis in bacteria and has been reported to be important in the process by which bacteria resist plant defense responses such as PAMP-related reactive oxygen species (ROS)-triggered immunity (PTI)(Khan et al., 2019).

Conclusively, our results showed that there were significant differences between healthy, diseased, and biocontrol agent-treated rhizosphere soil samples. The microbiota of tobacco changed dramatically in terms of the diversity index and taxa composition. Differentially abundant genera were identified by heatmap and LEfSe analyses and confirmed by network analysis. Functional predictions also revealed the beneficial and recovery potentials of healthy and biocontrol agent-treated samples, respectively. These findings will improve our knowledge of plant-microbe interactions and the application of biocontrol agents to improve plant fitness, and may contribute to the selection of biocontrol strains. However, further greenhouse experiments are required to confirm the present results.

## Data availability statement

The datasets presented in this study can be found in online repositories. The names of the repository/repositories and accession number(s) can be found below: https://www.ncbi.nlm.nih.gov/, PRJNA902686.

## Author contributions

H-LW conceived the study and supervised the project. Y-LG, JL, YZ and ZX performed the field experiments and collected samples. H-LW, Y-LG and Y-NM revised the manuscript. Y-NM and XW conducted informatics analysis. Y-NM wrote the manuscript. All authors have read and approved the final manuscript. All authors contributed to the article and approved the submitted version.
