# Supplementary material for: Deciphering the rhizosphere bacteriome associated with biological control of tobacco black shank disease
Source: Front Plant Sci. 2023 Apr 3;14:1152639. doi: 10.3389/fpls.2023.1152639 (PMC10108594; doi:10.3389/fpls.2023.1152639)
Supplement: Supplementary file 2 [file DataSheet_1.pdf]

Application of a biocontrol agent modulates the bacterial assemblage in the rhizosphere of tobacco infected with black shank disease

Yi-Nan Ma[a], Yi-Lin Gu[a], Jing Liu[c], Yuqin Zhang[d], Xinwei Wang[e], Zhenyuan Xia[b#] and Hai-Lei Wei[a#]

[a]Key Laboratory of Microbial Resources Collection and Preservation, Ministry of Agriculture and Rural Affairs, Institute of Agricultural Resources and Regional Planning, Chinese Academy of Agricultural Sciences, Beijing 100081, China

[b]Yunnan Academy of Tobacco Agricultural Science, Kunming 650021, China

[c]Zunyi Tobacco Company of Guizhou Provincial Tobacco Corporation, Zunyi 563000, China

[d]China National Tobacco Corporation Shandong Branch, Jinan 250101, China

[e]Tobacco Research Institute of Chinese Academy of Agricultural Sciences, Qingdao 266101, China

Running title: Tobacco rhizosphere microbiome

[#]Corresponding authors:

Zhenyuan Xia

Yunnan Academy of Tobacco Agricultural Science, 33 Yuantong St, Kunming 650021, China

Tel: 86- 871-65176766; E-mail: weihailei@caas.cn

Hai-Lei Wei

Key Laboratory of Microbial Resources Collection and Preservation, Ministry of Agriculture and Rural Affairs, Institute of Agricultural Resources and Regional Planning, Chinese Academy of Agricultural Sciences, 12 Zhongguancun South St, Haidian District, Beijing 100081, China

Tel: 86-10-82106243; E-mail: weihailei@caas.cn

The authors declare no conflict of interest.

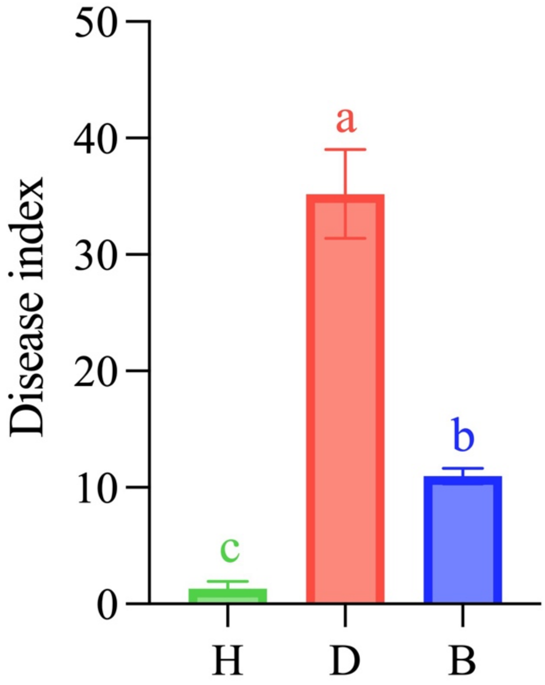

Supplementary Fig 1. **Disease index of H, D and B group tobacco samples.** Samples were collected about 8 weeks after the transplanting. Statistically significant differences (One-way ANOVA) were calculated between different soil samples. Lower letters represent statistical differences at the 95% confidence interval ($p < 0.05$).

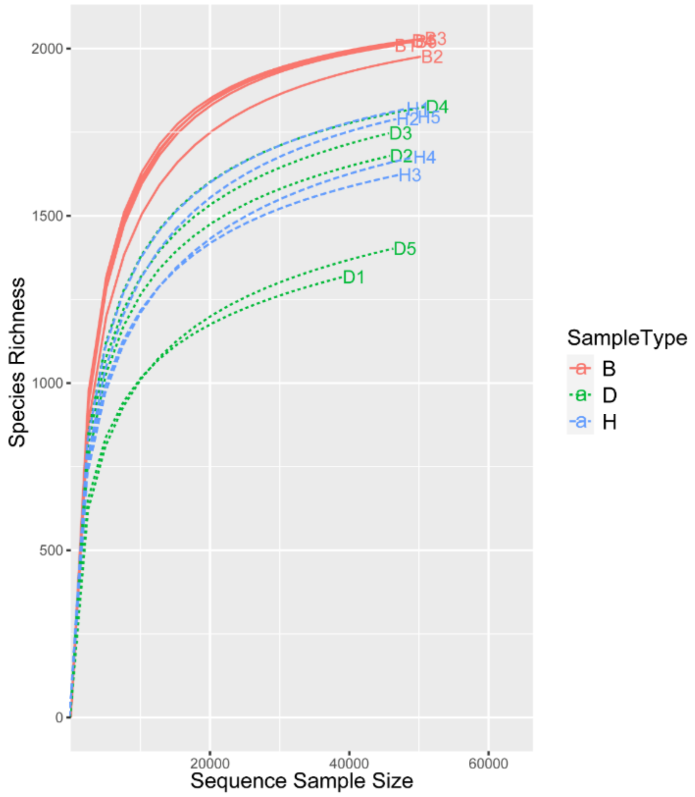

Supplementary Fig 2. Rarefaction curves of 15 samples from three groups.

Rarefaction curves are generated showing the number of ASVs using 100 steps as rarefaction calculations, relative to the number of total sequences. All the samples were rarefied to the sample with the least sequences before downstream analyses.

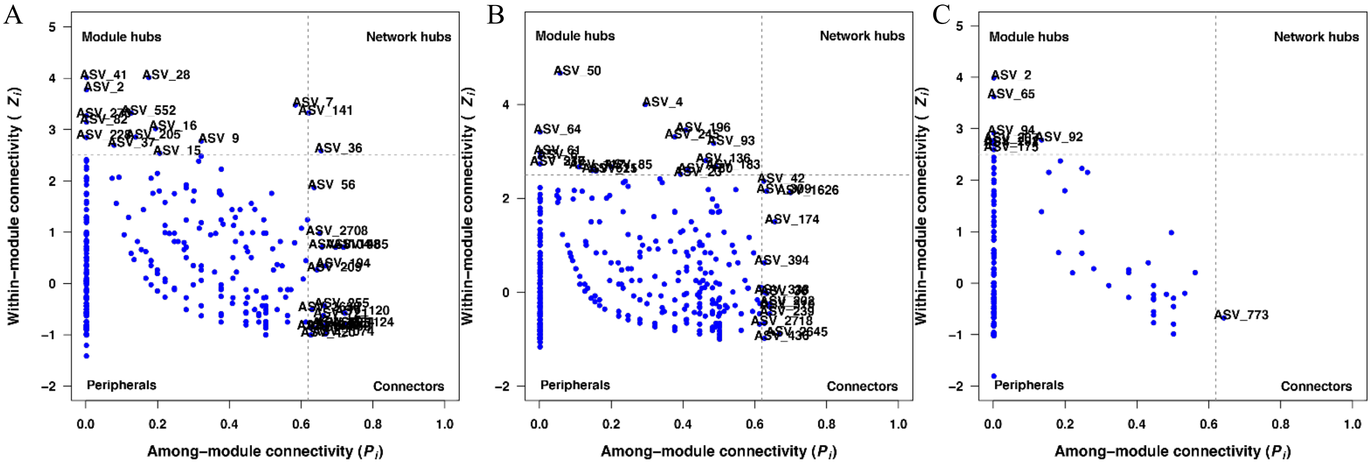

Supplementary Fig 3. *Zi-Pi* plots showing the distribution of ASVs based on their topological roles in the healthy (A), disease (B) and biocontrol agent (C) group-based networks. The within-module connectivity (*Zi*) and among-module connectivity (*Pi*) of each node were calculated to classify putative keystone ASVs in the network. Node topologies were organized into four categories: module hubs (highly connected nodes within modules, *Zi* > 2.5), network hubs (highly connected nodes within the entire network, *Zi* >2.5 and *Pi* > 0.62), connectors (nodes that connect modules, *Pi*> 0.62), and peripherals (nodes connected within modules with few outside connections, *Zi* < 2.5 and *Pi* < 0.62)
